# Both ANT and ATPase are essential for mitochondrial permeability transition but not depolarization

**DOI:** 10.1016/j.isci.2022.105447

**Published:** 2022-10-28

**Authors:** M.A. Neginskaya, S.E. Morris, E.V. Pavlov

**Affiliations:** 1 Department of Molecular Pathobiology, New York University, 345 East 24th Street, New York, NY, USA

**Keywords:** Molecular biology, Cell biology, Functional aspects of cell biology

## Abstract

An increase in permeability of the mitochondrial inner membrane, mitochondrial permeability transition (PT), is the central event responsible for cell death and tissue damage in conditions such as stroke and heart attack. PT is caused by the cyclosporin A (CSA)-dependent calcium-induced pore, the permeability transition pore (PTP). The molecular details of PTP are incompletely understood. We utilized holographic and fluorescent microscopy to assess the contribution of ATP synthase and adenine nucleotide translocator (ANT) toward PTP. In cells lacking either ATP synthase or ANT, we observed CSA-sensitive membrane depolarization, but not high-conductance PTP. In wild-type cells, calcium-induced CSA-sensitive depolarization preceded opening of PTP, which occurred only after nearly complete mitochondrial membrane depolarization. We propose that both ATP synthase and ANT are required for high-conductance PTP but not depolarization, which presumably occurs through activation of the low-conductance PT, which has a molecular nature that is different from both complexes.

## Highlights

• Both ATP synthase and ANT are required for permeabilization, but not depolarization
• CSA-dependent depolarization precedes mitochondrial permeabilization
• CSA-dependent depolarization is not dependent on ANT or ATP synthase
• Mitochondria disappear from holographic images upon membrane permeabilization

## Graphical abstract

**Figure:**
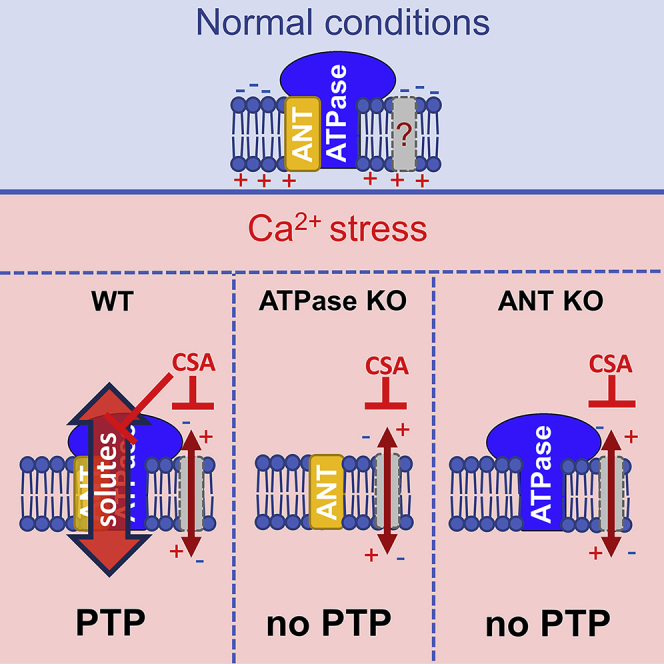

## Introduction

The low permeability of the mitochondrial inner membrane is an essential condition for efficient coupling between respiratory chain activity and phosphorylation of ADP by ATP synthase.^1^^,^^2^ An increase in the permeability of the inner membrane leads to mitochondrial membrane depolarization, uncoupling of the oxidative phosphorylation, and mitochondrial energy failure. It is generally accepted that stress-induced increase in the permeability of the mitochondrial inner membrane, known as permeability transition (PT), is a critical contributor toward cell death in a wide range of pathologies associated with hypoxic-ischemic injuries.^3^ PT is caused by the activation of the PT pore (PTP) in the mitochondrial inner membrane. The signature feature of PTP is an unselective increase in membrane permeability to ions and other molecules up to 1.5 kDa in size, which can be blocked by cyclosporin A (CSA).^4^ The molecular mechanisms of PTP are not entirely understood and are the subject of intensive investigation and considerable controversies.^5^^,^^6^^,^^7^^,^^8^^,^^9^^,^^10^^,^^11^ Genetic knockout studies suggest that PTP involves both adenine nucleotide translocator (ANT) and ATP synthase (ATPase).^12^^,^^13^ However, both ANT^14^ and ATPase^5^^,^^11^^,^^15^^,^^16^ can form the pore when purified from the mitochondria and reconstituted into model membranes. Taking into account that in the native membranes multiple channel conductance’s have been identified,^17^ the question about which of these channels is responsible for PTP formation remains open.

**Figure 1:**
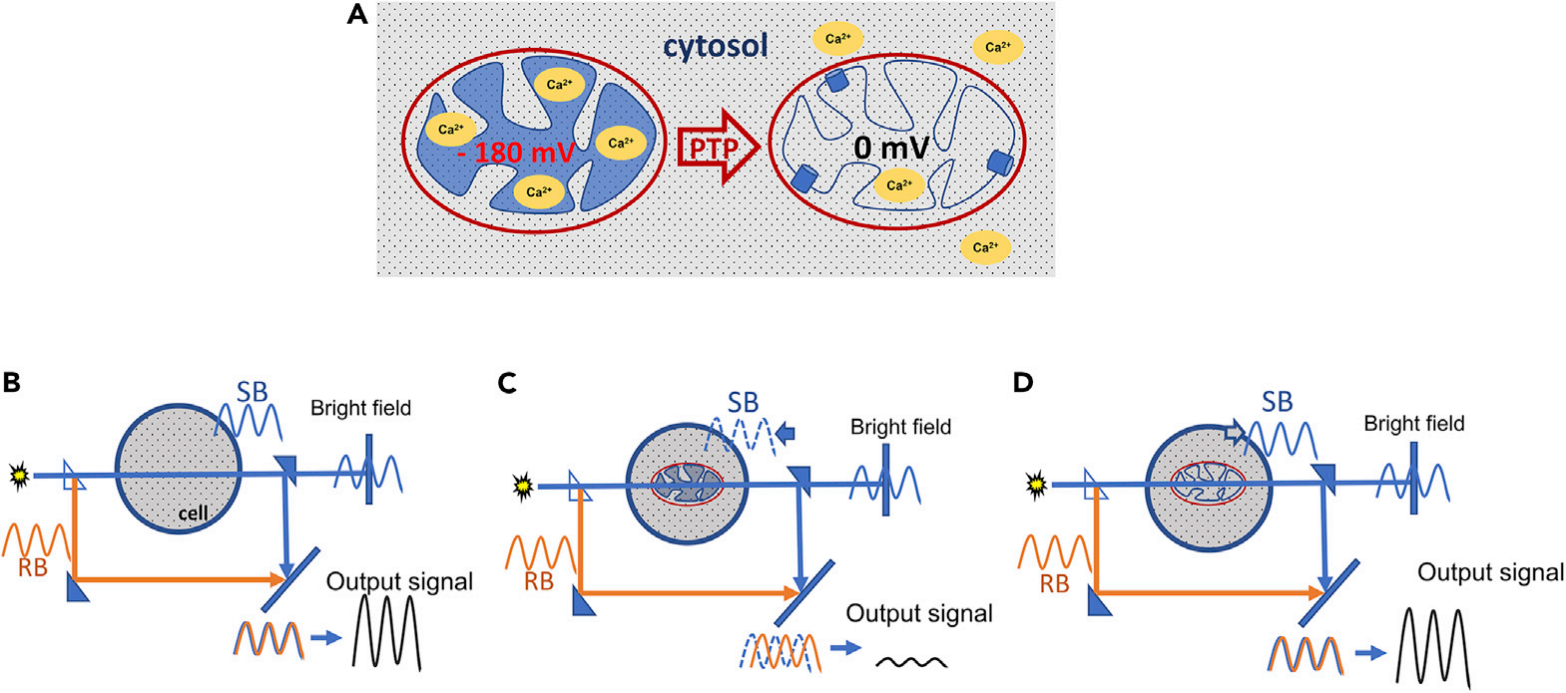
Principles of mitochondrial permeability transition assay in living cells using holographic microscopy (A) PTP causes the equalization of RIs between mitochondrial matrix and cytosol. Mitochondrial Ca^2+^ overload leads to the induction of the mitochondrial membrane permeabilization that occurs due to the opening of PTP in the inner mitochondrial membrane. PTP opening causes equilibration of solutes up to 1.5 kDa in size between matrix and cytosol. Equilibration of solutes results in equalization of RIs (and Optical Densities, ODs) between mitochondrial matrix and cytosol. (B) Principles of the holographic imaging. A conventional bright-field microscope detects only the signal that is passed through the sample, the sample beam (SB). Holographic imaging detects the interference pattern of 2 light beams: the reference beam (RB) and SB, which passes through the object of interest. SB is initially equal to RB but becomes delayed by passing through the sample. This delay of SB depends on the RIs of the content inside the sample. In the present example, SB and RB reach the detector in the same phase, and the holographic microscope detector registers the interference, which will be the sum of two beams with equal phase and amplitude. (C) Intact mitochondrion has different RI (higher OD) when compared to the cytosol of the cell. If cellular cytosol contains intact mitochondrion with higher OD, the SB is delayed due to the slower light speed inside the matrix compared to the RB. As a result, the interference pattern will be changed because of the differences between the phases of RB and SB. A bright-field microscope cannot distinguish between SB with or without the mitochondrion, as the mitochondrion is transparent and does not cause enough of decrease in intensity of the light. (D) Following the opening of PTP, the RI (and OD) of the mitochondrial matrix is equalized with the RI of the cell cytosol and there is no delay of SB. In this case, the interference pattern detected by the microscope will be the same as that of the cytosol without mitochondrion (compare outcome signal in panel B). As a result, mitochondrion with PTP opened becomes “invisible” to the holographic microscope.

We reasoned that such a controversy could be explained by the lack of unambiguous methodology to measure PT inside the living cells. Despite the large arsenal of methods available to experimentally study PTP, the number of direct assays in the intact cells is surprisingly limited, with many conclusions regarding PTP activity derived from the measurements of mitochondrial membrane depolarization.^6^ Since depolarization is not necessarily caused by the PTP, this method often leads to inconclusive results and interpretations. To overcome this problem, we developed an assay that is based on the technique of holographic imaging.^18^ This assay allows direct detection of mitochondrial membrane permeabilization (and hence PTP) inside the living cells. A holographic microscope generates images based on the differences in refractive indexes (RI) of the object parts. RI reflects how fast the light propagates through the object (mitochondrion in the case of this study). By estimation of the delay of the light passing through the matrix of the intact mitochondria with higher RI, the holographic microscope can reconstruct their shape (Figure 1). Due to the large size of PTP, the immediate consequence of its opening is a rapid exchange of solute contents across the mitochondrial inner membrane. This exchange causes equilibration of the solute content and results in the equalization of RI between the mitochondrial matrix and its surroundings (Figure 1C) and as a result, disappearance of the organelles from holographic image (RI image).

Here, we use a combination of fluorescent and holographic microscopy to simultaneously measure the changes of mitochondrial membrane potential and PTP activation in wild-type cells, as well as in cells lacking either ATPase or ANT.

We discovered that calcium-induced high-conductance PTP is preceded by the initial stage of membrane depolarization (which we define as low-conductance permeability transition). Furthermore, we demonstrate that deletion of either ATPase or ANT leads to complete elimination of PTP. Interestingly, neither of these proteins was essential for CSA-sensitive calcium-induced loss of membrane potential. We hypothesize that activation of PTP requires cooperative molecular interactions of ANT and ATPase.

## Results

### Visualization of mitochondria inside the living cells

**Figure 2:**
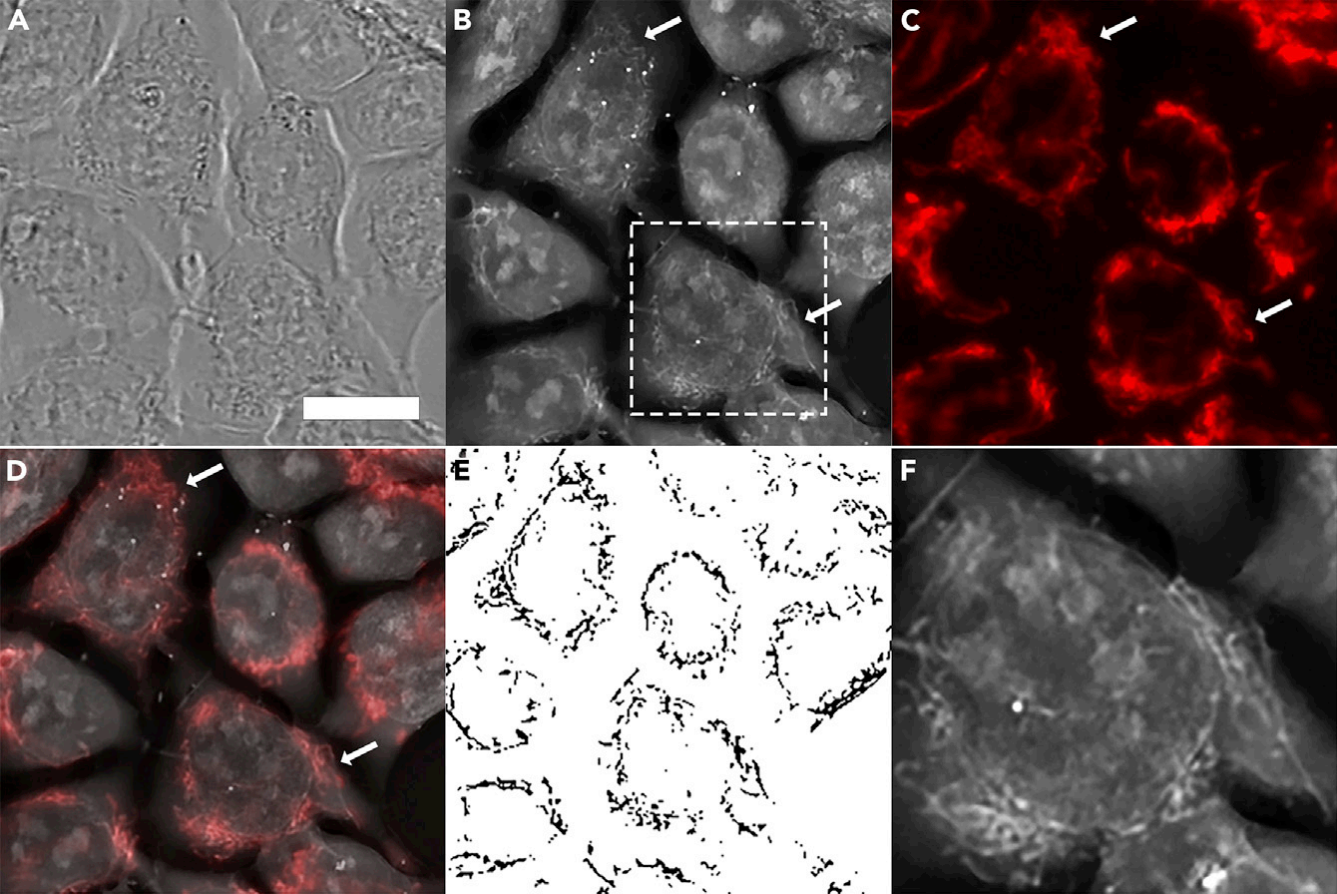
Holographic imaging allows for label-free monitoring of mitochondria in the living cells (A) Bright-field image of immortalized HAP-1 cell culture. (B) Holographic image of the same area of the cells. No label was used. (C) Staining of mitochondria with membrane potential sensitive probe TMRM. Areas with live mitochondria that maintain membrane potential are visible in fluorescent microscope. Arrows point to mitochondrial areas. (D) Overlay, of the images shown on panels B and C showing colocalization between RI (B) and TMRM images (C). (E) Segmentation of the mitochondrial regions from the holographic image shown on panel B. These segmented images were used for quantification of the PTP activation. (F) Enlarged images of the region shown in the white box on panel B. Scale bar: 10 μm for A–E; 5 μm for F.

In a holographic image, the contrast is achieved based on the differences in the RI of different areas of the cell.^19^ Figure 2 illustrates that holographic imaging allows for the distinguishing of inner cellular structures that are not visible when bright-field imaging is applied (compare images on Figures 2A and 2B). As shown in the RI image in Figures 2B and 2F, mitochondria (arrows) are visible directly inside the living cell without the use of fluorescent labels. The identity of these structures was confirmed by the fluorescent probe TMRM, which selectively labels polarized mitochondria (Figure 2C). Overlay of the RI and TMRM images allowed us to clearly identify structures representing mitochondria (Figure 2D). Using a segmentation tool, we convert holographic images to binary mitochondrial maps (Figure 2E). These maps were used to track permeabilization of mitochondria following treatments.

### Ferutinin models mitochondrial PT

We used calcium ionophore ferutinin (C_22_H_30_O_4_, Figure S1) to model PT conditions. It has been demonstrated that calcium can bind to ferutinin and, in such form, can cross the bilayer membrane due to the lipophilic properties of ferutinin.^20^ In isolated mitochondria, it has been shown that ferutinin leads to the accumulation of calcium in the mitochondrial matrix in a way that is independent of the mitochondrial calcium uniporter (MCU), leading to activation of PTP.^21^ In the intact cells, ferutinin was shown to electrogenically deliver calcium into mitochondria and induce calcium overload followed by CSA-sensitive mitochondrial depolarization,^22^ representing a robust cell culture model for the investigation of the molecular details of PTP.^6^^,^^7^^,^^23^^,^^24^

**Figure 3:**
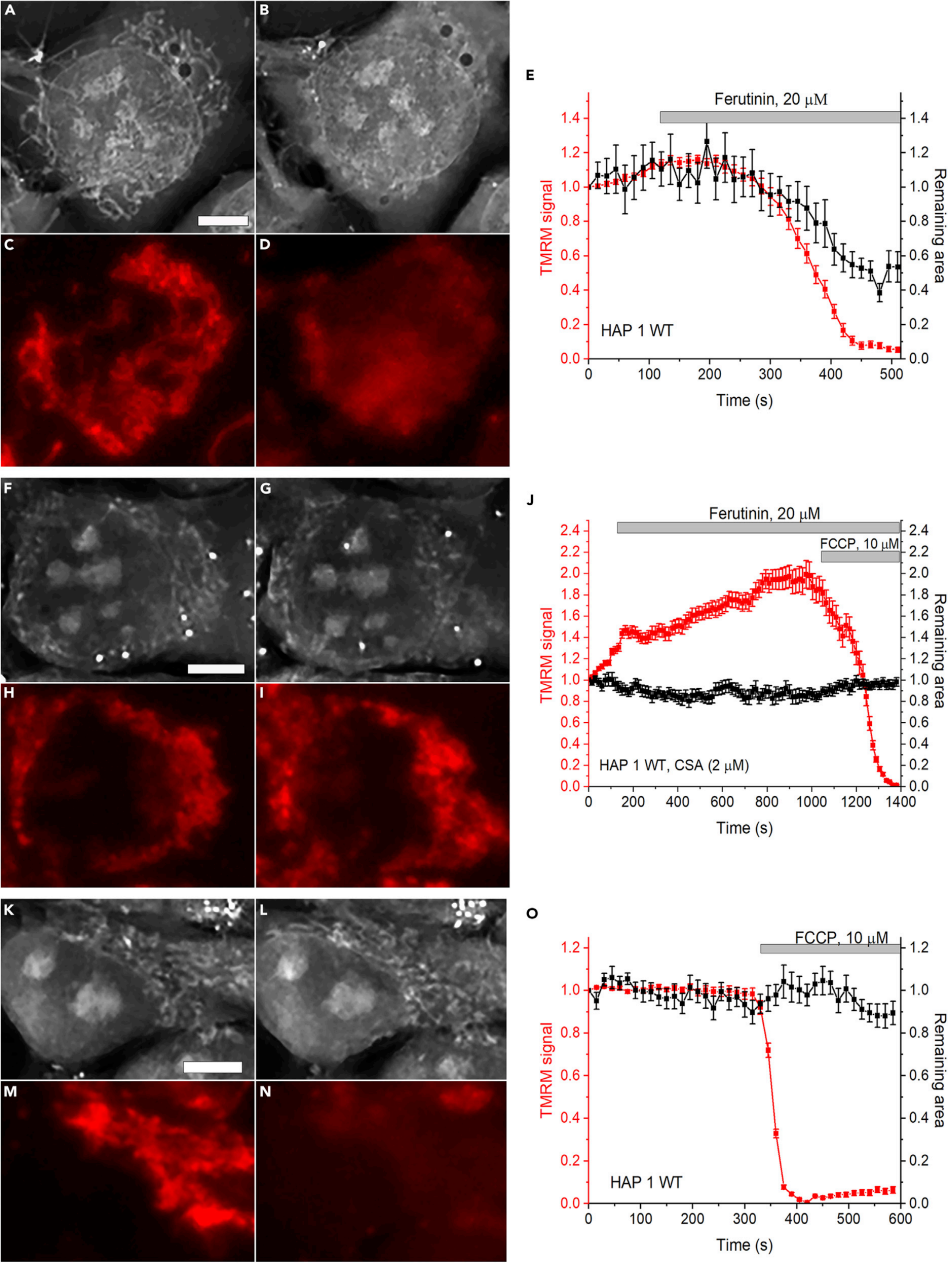
Simultaneous detection and quantification of mitochondrial depolarization (TMRM, red) and permeabilization (RI) in HAP 1 WT living cells (Panels A and B) show holographic images of the cells before and 6 min after the addition of ferutinin (20 μM). Note the disappearance of the mitochondria in panel B. (Panels C and D) show fluorescent imaging of the mitochondrial membrane potential from the same field as in images A and B. (Panels F–I), the same as in panels A–D but in the presence of CSA. (Panels K–N) – the same as in panels A–D but with only the addition of FCCP. (E, J, O) time-dependent changes of TMRM and RI signals of the mitochondrial regions. Representative experiments of N = 5 for Ferutinin, N = 4 for Ferutinin + CSA, N = 3 for FCCP. Each data point on the traces represents mean ± SEM calculated from different mitochondrial regions. Scale bar – 5 μm.

**Figure 4:**
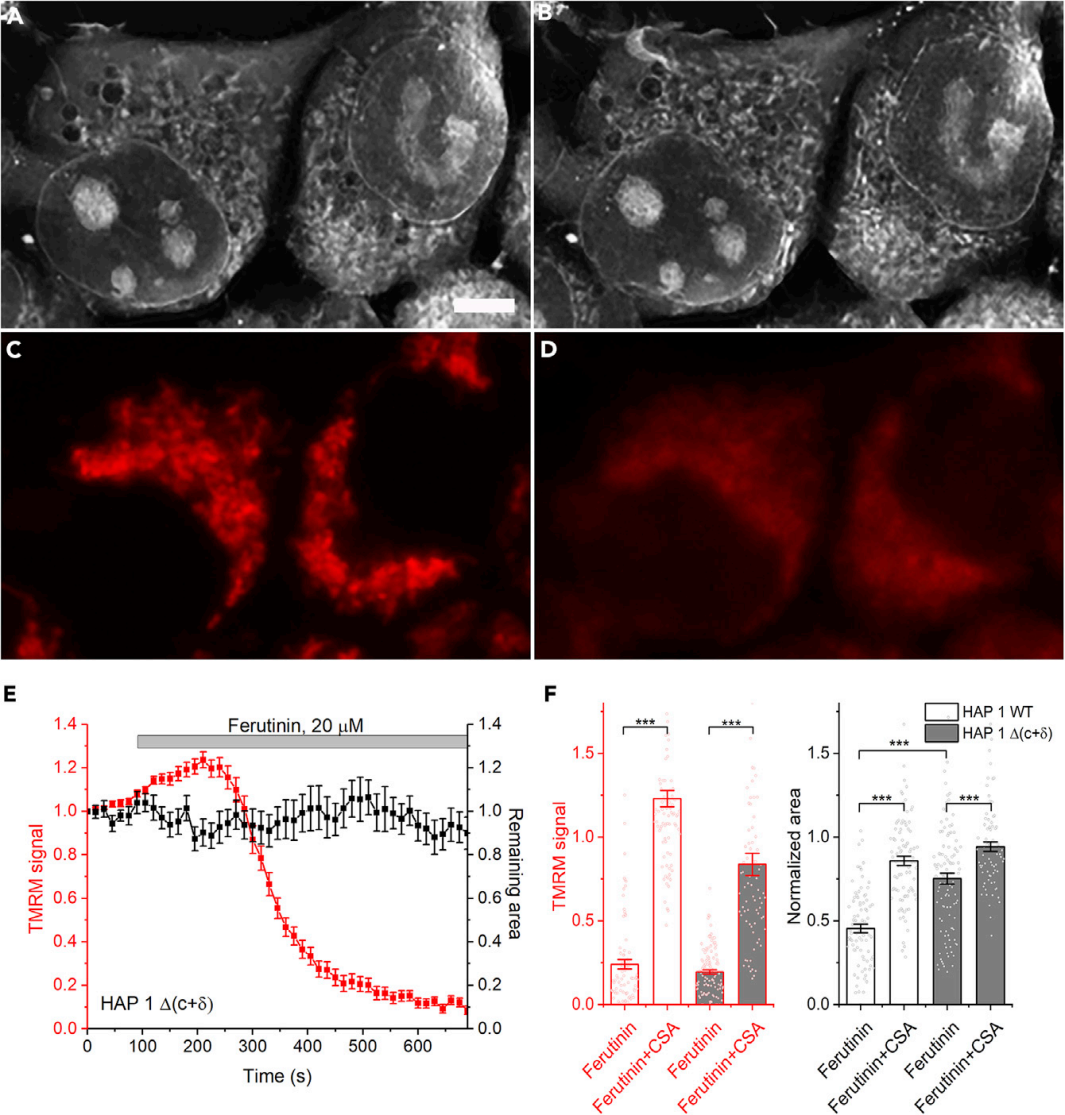
Lack of high-conductance PTP despite membrane depolarization in HAP 1 Δ (c+δ) cells (A–D) Holographic and fluorescent (TMRM) images of cells before (A, C) and after (B, D) the addition of ferutinin (20 μM). Scale bar – 5 μm. (E) Time dependence of the membrane depolarization and refractive index measurements. (F) Quantification of the degrees of membrane depolarization and permeabilization. TMRM signal after ferutinin addition in presence or absence of CSA (2 μM). Corresponding raw values of TMRM signal presented on Figure S4, panel B. Mean ± SEM; One way ANOVA; ∗∗∗p < 0.001.

We measured the response of mitochondria to the addition of ferutinin in HAP 1 WT cells by simultaneously monitoring the membrane potential and RI of the mitochondria (Figures 3A–3E and 4F). As can be seen from the figures, activation of PTP with ferutinin (20 μM) leads to mitochondrial depolarization (Figures 3C and 3D) and disappearance of the mitochondrial structures from the RI images (Figures 3A and 3B), which is consistent with the equilibration of the solutes and thus optical densities (and RI) between the matrix and cytoplasm. By segmenting mitochondria from other cellular structures and conversion of RI images to binary images (Figure S2), we were able to track the drop in RI/“disappearance” of mitochondria with PTP as a decrease of the mitochondrial area on binary images (Figure 3E, black trace; Figure S2). Drop in RI of mitochondria coincided with membrane depolarization that was detected by the decrease in TMRM signal (Figures 3E and 4F; N = 5; n = 82). Both membrane depolarization and RI drop were prevented by the addition of CSA (Figures 3F–3J and 4F; N = 4; n = 89). These data demonstrate that non-selective mitochondrial membrane permeabilization can be directly detected in the living cells and that this increase in permeability is indicative of the activation of the CSA-sensitive high-conductance PTP. In control experiments, when depolarization was induced by the protonophore FCCP without PTP induction, despite the drop in TMRM fluorescence, mitochondria remained intact and easily recognizable in the RI images (Figures 3K–3O and S3; N = 3; n = 55) demonstrating that the detection of permeabilization does not depend on membrane potential.

### Cells lacking assembled ATP synthase undergo CSA-sensitive depolarization but not membrane permeabilization

It has been suggested that ATPase plays an important role in the PTP. However, recent studies using a double knockout HAP 1 mutant (HAP 1 Δ (c+δ)), lacking c and δ subunits and, consequently, making them devoid of the assembled ATPase, show that these mitochondria can still undergo calcium-induced depolarization in the intact cells when stimulated with ferutinin.^6^ Using holographic imaging, we investigated the relationship between the calcium-induced depolarization and high-amplitude mitochondrial permeabilization. As demonstrated in Figure 4, unlike wild-type cells, HAP 1 Δ (c+δ) cells did not undergo high-amplitude permeabilization, despite membrane depolarization (Figure 4; N = 5, n = 108). This suggests that the assembled ATP synthase is required for the development of the high-conductance PTP but is not involved in the initial membrane depolarization that is triggered by the addition of ferutinin. Importantly, this initial depolarization was inhibited by CSA in both WT and HAP 1 Δ (c+δ) (Figure 4F), suggesting that it represents one of the stages of the PTP activation process. Interestingly, disassembled ATPase in HAP 1 Δ (c+δ) resulted in lower basal membrane potential when compared to HAP 1 WT cells. However, mitochondria in HAP 1 Δ (c+δ) preserved the ability to depolarize in a CSA-sensitive manner after ferutinin addition (comparison of raw TMRM signals in HAP 1 WT and HAP 1 Δ (c+δ) presented in Figure S4).

**Figure 5:**
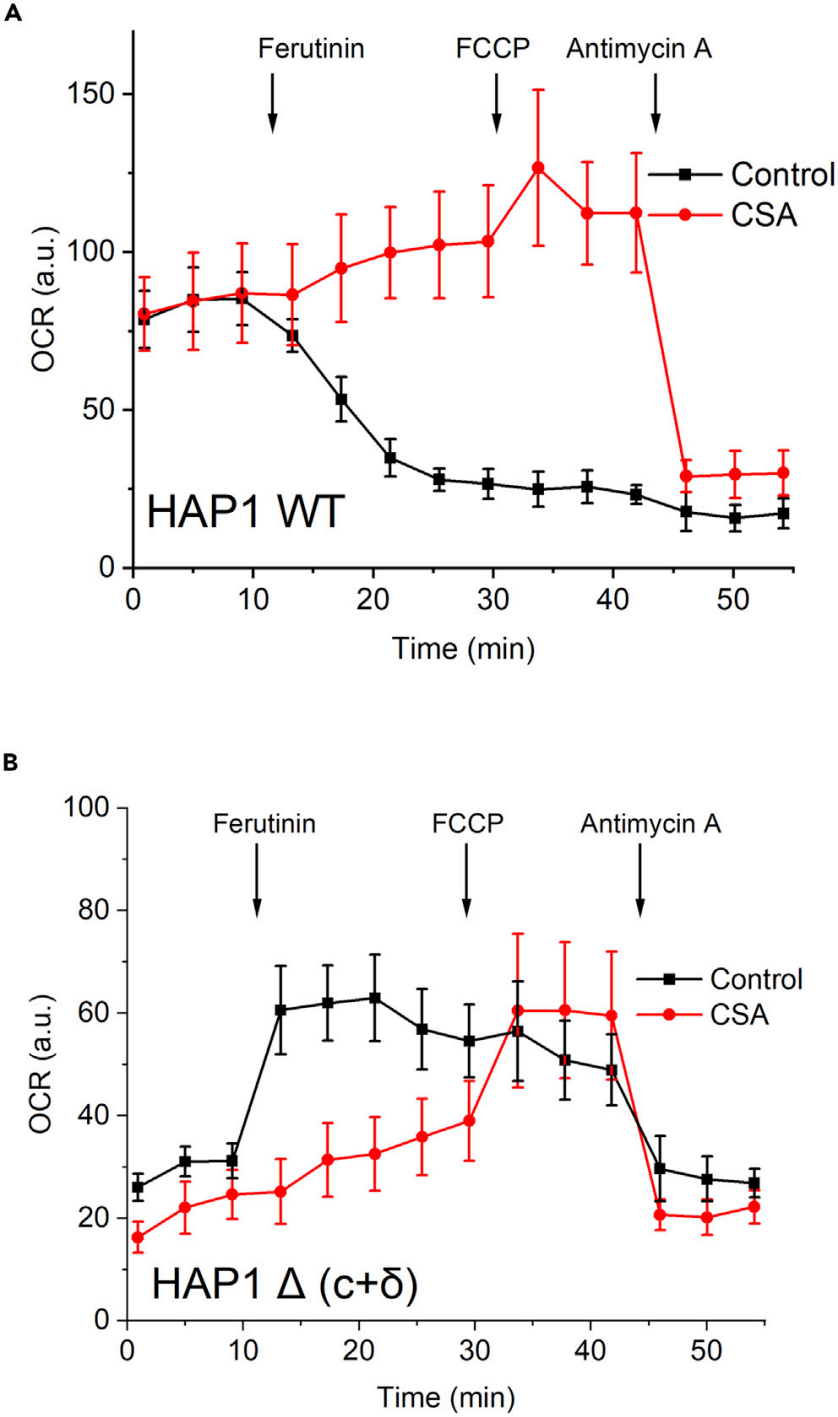
Seahorse analysis of the oxygen consumption rates changes in response to ferutinin (30 μM) (A) HAP 1 WT cells demonstrated a dramatic decrease in respiratory rates that was prevented by CSA (2 μM) consistent with the loss of mitochondrial function due to the PTP. (B) HAP 1 Δ (c+δ) cells respiration was stimulated by ferutinin (30 μM) but was not further stimulated by the uncoupler FCCP, consistent with the stimulation of the mitochondrial respiration due to Ca^2+^-induced uncoupling without high-amplitude PTP opening. This stimulation was prevented by CSA (2 μM). Data are representative of 3 independent experiments. Each data point on the traces represents mean ± SEM.

The lack of high-amplitude permeabilization was further confirmed by measuring the effects of ferutinin on mitochondrial respiration using the Seahorse metabolic flux analyzer. As can be seen from Figure 5A, ferutinin caused rapid loss of mitochondrial function in the WT cells consistent with what would be expected from the high-conductance PTP activation and loss of the respiratory chain substrates. On the contrary, the same amount of ferutinin transiently stimulated mitochondrial respiration in the HAP 1 Δ (c+δ) cells (Figure 5B). This stimulation of the respiration is consistent with the observation that despite depolarization, mitochondria of these mutant cells remained structurally intact which allowed them to (at least transiently) maintain respiratory activity. The effects of the addition of ferutinin on the respiratory function for both cell types were blocked by CSA (Figure 5), confirming that both processes, membrane depolarization and mitochondrial permeabilization, are related to PTP.

### Cells lacking ANT undergo CSA-sensitive depolarization but not membrane permeabilization

**Figure 6:**
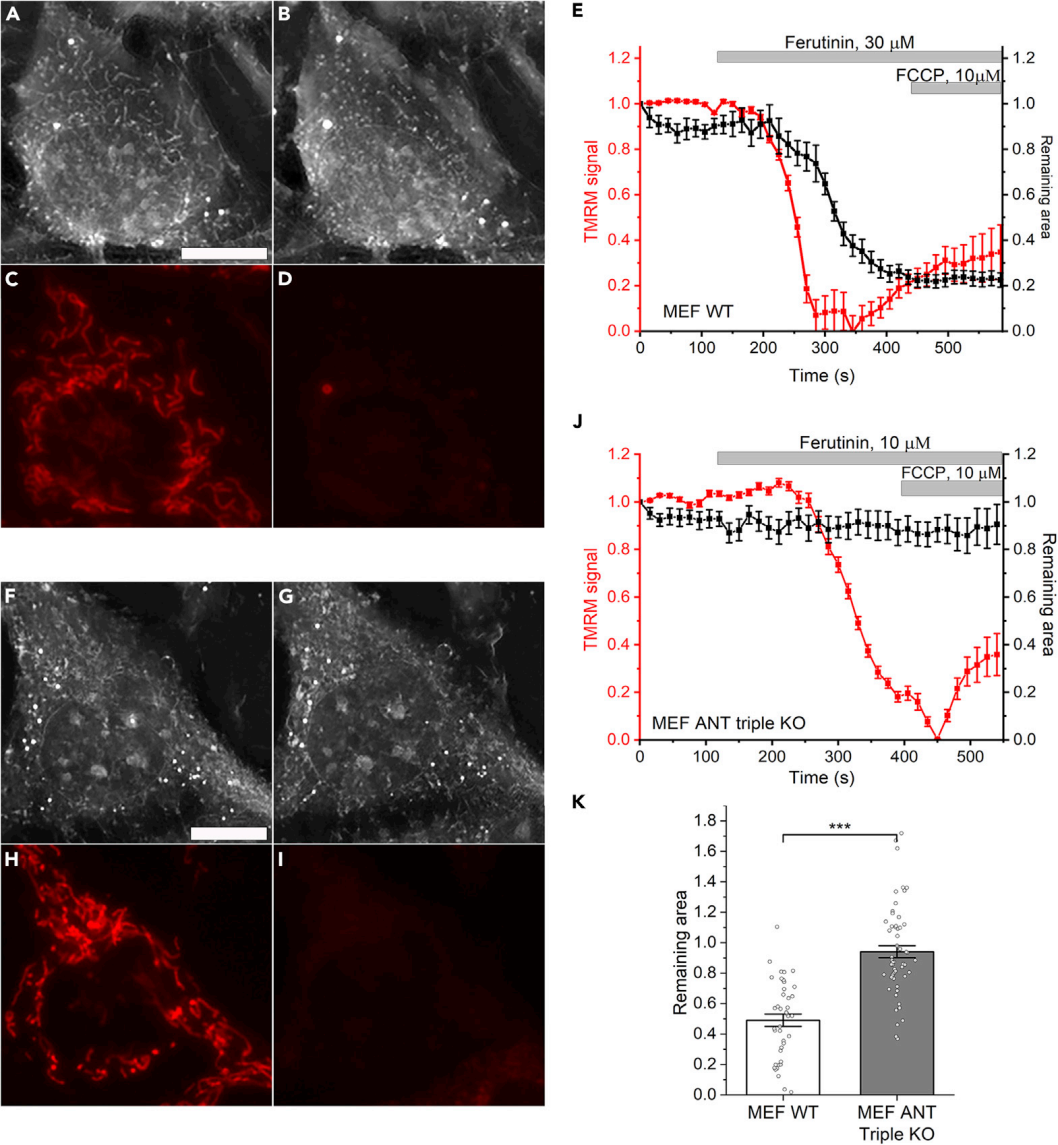
Lack of high-conductance PTP in MEF ANT triple KO cells (A–D) Simultaneous imaging of the RI and TMRM fluorescence before (A and C) and after addition of ferutinin (B and D) in MEF WT cells. (F–I) RI and TMRM fluorescence before (F and H) and after addition of ferutinin (G and I) to ANT triple KO MEF cells. Note that despite mitochondrial depolarization, mitochondria are still visible on RI images. (E and J) time resolved quantification of the RI and TMRM signals following the addition of ferutinin. (K) statistical analysis of the remaining mitochondrial area in RI images following ferutinin-induced depolarization. Note that mitochondria did not disappear in MEF ANT triple KO cells. N = 4 and n = 41 for WT; N = 5 and n = 56 for KO; Mean ± SEM; One way ANOVA; ∗∗∗p < 0.001.

Next, using holographic assay, we checked the permeabilization of mitochondria inside the mouse embryonic fibroblasts (MEF) WT and MEF ANT triple KO cells upon ferutinin addition (30 μM for WT and 10 μM for ANT triple KO cells). Previous experiments showed that MEF ANT triple KO cells have significantly inhibited PTP.^8^ In MEF WT cells, decrease of the area occupied by mitochondria (Figures 6A, 6B, and 6E) followed the depolarization induced by ferutinin (30 μM) addition (Figures 6C–6E; N = 4; n = 41; Figure S5A). Like in case of HAP 1 cells, this process was inhibited by CSA, suggesting the involvement of PT (Figures S5B and S5C). However, in MEF ANT triple KO cells, we did not observe any significant reduction in the area occupied by mitochondria followed by ferutinin (10 μM) treatment, while we still observed a dramatic loss of membrane potential (Figures 6F–6J; N = 5; n = 56). Comparison of remaining mitochondrial area after ferutinin addition in WT and ANT triple KO cells is shown on Figure 6K. These results suggest that like ATP synthase, ANT is also essential for the development of high-conductance PTP but not involved in calcium-induced CSA-sensitive mitochondrial depolarization.

### Mitochondrial calcium overload induces mitochondrial depolarization that precedes high-conductance permeabilization

**Figure 7:**
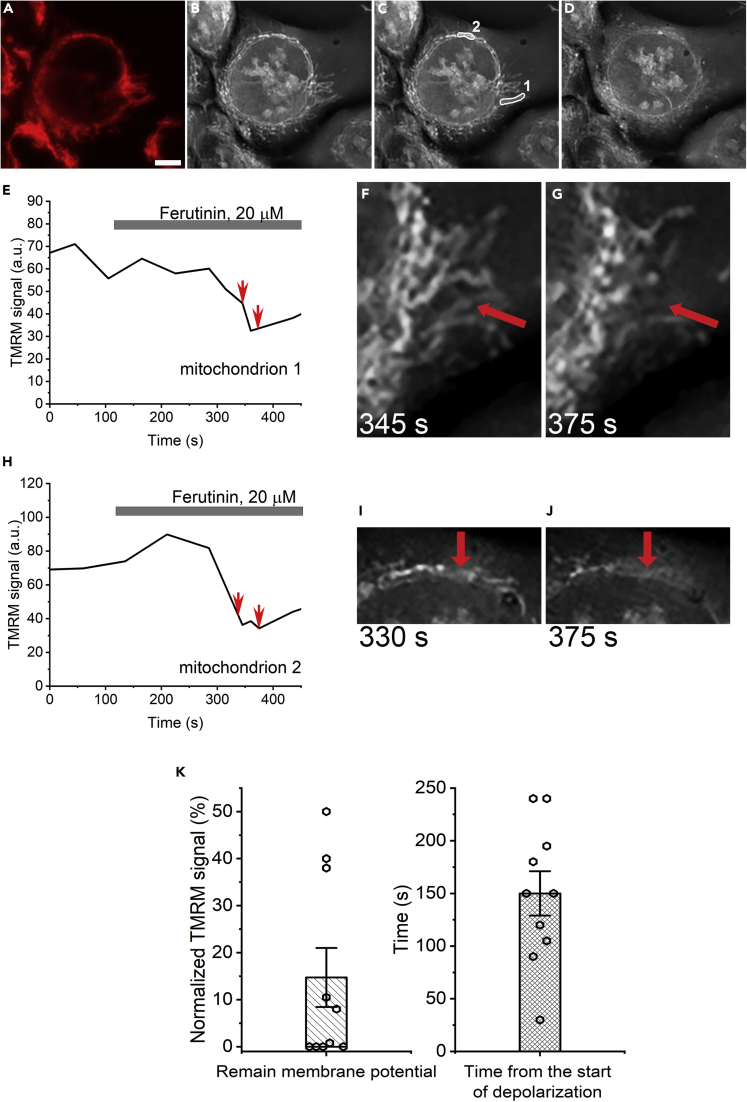
Monitoring time-dependent membrane depolarization and high-amplitude permeabilization in HAP 1 WT cells at the level of a single mitochondrion (A–D) Fluorescent (TMRM) (A) and holographic images of the cell at the beginning of the experiment (B and C) and following ferutinin addition (D). Labels on panel C show the selection of two representative mitochondria. Scale bar – 5 μm. (E and H) time dependence of the TMRM fluorescence from the mitochondria #1 and #2 (see panel C). (F and G) images correspond to the time points marked by arrows at panel E. For mitochondrion #1; note the disappearance of mitochondrion from the panel G. (H–J) analysis similar to that of panels E-G for mitochondrion #2. (K) The relationship between mitochondrial depolarization and permeabilization at the level of the single mitochondrion. Left panel, the level of the residual membrane potential at the moment of mitochondrial permeabilization (n = 10). Right panel, the time delay between the offset of depolarization and permeabilization (n = 10; p < 0.001; t-test for null-hypothesis). Mean ± SEM.

In both mutant cell lines, despite the lack of high-conductance PTP, we observed mitochondrial depolarization. To gain further insight into the relationship between depolarization and permeabilization, we analyzed the dynamics of these two processes at the single mitochondrion level. Figure 7 shows the result of simultaneous analysis of the dynamics of mitochondrial membrane potential and non-selective permeabilization performed at the level of a single mitochondrion. Here, we traced individual mitochondria using both RI and TMRM readouts from the moment before treatment where mitochondria were functional (Figure 7A) and visible on RI image (Figure 7B) until the mitochondrial disappearance (Figure 7D). The specific organelles RI were tracked throughout the duration of the experiment frame by frame as shown in Figure 7C for 2 selected mitochondria. TMRM signal was detected at corresponding areas of fluorescent images (Figure 7A). As shown in Figures 7E and 7H, ferutinin caused a gradual decrease in the membrane potential. Interestingly, despite significant membrane depolarization, the RI of individual mitochondrion stayed largely undisturbed and individual mitochondrion remained clearly visible (Figures 7E and 7F for mitochondrion 1, and 7H and 7I for mitochondrion 2). However, mitochondria rapidly disappeared from RI images when depolarization was nearly complete (Figures 6E and 6G for mitochondrion 1, and 6H and 6J for mitochondrion 2). The membrane potential of individual mitochondrion at the moment of organelle disappearance from the RI image was 15 ± 6% of the initial potential level (Figure 7K, left panel, n = 10). The average time delay from the start of depolarization until the disappearance of the individual mitochondrion was 150 ± 20 s (Figure 7K, right panel, n = 10; p < 0.001). Overall, individual mitochondrion analysis showed that almost complete depolarization occurred prior to the onset of non-selective large-scale membrane permeabilization, and on average, the permeabilization was delayed by 150 ± 20 s from the beginning of the depolarization (Figure 7K, n = 10; p < 0.001). Altogether, these experiments indicated that initial depolarization occurred prior to high-conductance PTP activation. This is a new insight that suggests that the high-conductance PTP is not the cause of membrane depolarization.

## Discussion

Traditionally, a functional assay of the PTP in intact cells relies on fluorescent measurements of the mitochondrial parameters. In most cases, PTP can be experimentally identified as a calcium-induced CSA-sensitive membrane depolarization and/or calcium release, both of which can be detected fluorometrically in the intact cells.^25^^,^^26^ Notably, these methods do not necessarily indicate activation of the high-conductance PTP. To our knowledge, the only fluorescent method specifically geared toward PTP is monitoring of the calcein release from the mitochondria,^27^ where calcein release would indicate the opening of the large pore. However, calcium-triggered calcein (which is similar in size to the essential mitochondrial energy metabolite NADH known to be released through PTP) release can occur in a CSA-independent manner and without the loss of mitochondrial function,^27^ suggesting that in addition to PTP this release can proceed through the mechanisms independent of the simple size-exclusion diffusion through the large pore. The method described here provides a direct assay that relies on the definitive feature of PTP, which does not rely on tracking of the transport of the specific molecule but rather reflects the non-selective equilibration of the solutes across the mitochondrial membrane. Therefore, our method, when combined with calcein release or NADH fluorescent detection, should be able to provide new insights on the relationship between inner membrane permeabilization and calcium or NADH release.

Furthermore, unlike in experiments involving isolated mitochondria in the population, we were able to monitor optical density (or RI) in a single mitochondrion. This is an important advantage as changes in light scattering in the population of mitochondria might not necessarily reflect complete swelling of individual organelles, but rather gradual changes in “average” light scattering across the whole population. We anticipate that this method, with the help of genetically encoded fluorescent proteins, will clarify many details in the PT and mitochondrial swelling at the level of intact cells and resolve some current controversies. Another potential advantage of the high-resolution capability of this technique is that it would allow for the detection of localized PTP openings in such conditions as for example in the events of mitochondrial fission and fusion.^28^^,^^29^

One important aspect of PTP which this new method would allow for the clarification of, is the ability to more accurately estimate the relationship between PTP and mitochondrial swelling. It is known that, following calcium treatment, isolated mitochondria swell^30^^,^^31^ and, in the literature, generally the terms “light-scattering” and “swelling” assay are used interchangeably. However, prior to PTP opening, mitochondria are perfectly osmotically and oncotically balanced with the surrounding medium. Opening of the non-selective PTP—which allows flux of molecules of up to 1.5 kDa in size—would definitely cause a drop in RI due to the equilibration of the matrix and medium content. At the same time, however, this solute exchange should not necessarily lead to swelling in the living cell. The oncotic pressure of non-permeable proteins would remain balanced, as it was prior to PTP, while permeable molecules would exchange freely, leaving the net accompanying water flux unchanged. Single mitochondria RI imaging will help to clarify if swelling is indeed the direct consequence of the PTP opening, or if swelling occurs at the later stages of mitochondrion demise.

The advantage of being able to monitor RI in real-time with single organelle resolution is evident from our experiments with simultaneous monitoring of the RI in relation to the mitochondrial membrane potential. As shown in Figure 6, during the induction of the PTP by the addition of calcium, we detected that at the first stage mitochondria undergo membrane depolarization, followed by a second stage of the PTP characterized by high-amplitude membrane permeabilization. This observation challenges the widely accepted view that calcium-induced PTP is a cause of membrane depolarization.^32^ Our data suggest that the initial step of PTP activation is likely the opening of the lower conductance channel that is sufficient to depolarize mitochondria. This occurs prior to the activation of the high-conductance PTP which is required for mitochondrial swelling (as seen in the isolated mitochondria). Interestingly, previous studies on isolated mitochondria showed that the PTP channel is voltage dependent, more likely to be opened at lower voltages.^33^^,^^34^ Our study is consistent with the idea that membrane potential drop might be an important initial event that leads to the PTP opening.

The molecular mechanisms of PT activation and function remain incompletely understood. It is very likely that physically PT can occur through several pathways.^12^^,^^13^ One of the key challenges in the field is understanding the roles of the ATP synthase and ANT in this process. Compelling evidence from several independent laboratories supports competing interpretations suggesting that a core part of PTP involves either the ATP synthase complex or ANT, both of which could be transformed into the high-conductance pore.^5^^,^^11^^,^^14^^,^^15^^,^^16^^,^^35^ In both cell types lacking either ATPase or ANT, calcium treatment causes calcium release and membrane depolarization that is inhibited by CSA.^6^^,^^8^ Here, using the same knockout cell models, we observed the phenomena of membrane depolarization. However, holographic imaging revealed that mitochondria in these mutant cells did not undergo high-amplitude permeabilization. This suggests that both ATP synthase and ANT are essential for the development of the high-conductance PTP. These results are in agreement with the previously proposed model that, in fact, the functional PTP complex would require presence of both ATPase and ANT.^36^ The fact that none of these complexes are required for Ca^2+^-induced mitochondrial depolarization would explain the controversy in the literature regarding their roles in PTP. Furthermore, the requirement of the complex could explain the fact that very low number of pores are present in each individual mitochondrion despite the presence of many copies of ANT and ATPase.^37^^,^^38^^,^^39^

It is also tantalizing to suggest that the CSA-dependent depolarization step, that does not require permeabilization, is related to the phenomenon known from the literature as a low-conductance PTP.^40^ It has been suggested that low-conductance PTP could be beneficial to prevent mitochondria from calcium and ROS overload. Our findings suggest that the molecular nature of the low-conductance PTP might be distinctly different in nature from the high-conductance PTP. Interestingly, the low-conductance mode of PTP has been demonstrated to present in brain mitochondria following the condition of intermittent hypoxia in brain mitochondria.^41^^,^^42^ At present, it is difficult to suggest what mechanism might involve this mode, but it is possible that it can be provided by the opening of one of the mitochondrial ion selective channels or by leak mechanisms that are lipidic in nature.^43^^,^^44^^,^^45^^,^^46^^,^^47^

In summary, the two phenomena observed in our experiments suggest the presence of a low-conductance mode of PTP, which occurs independent of the ATP synthase and ANT, which are required for the high-conductance mode of PTP. We hypothesize that PTP development might be a two-channel phenomenon (ANT and ATPase) that demonstrate interdependence. The lack of PTP in cells lacking ATPase and ANT opens an exciting possibility that the two steps of PTP might involve different molecular structures. It is tantalizing to suggest that selectively targeting ATP synthase and ANT might help to identify compounds that would prevent mitochondrial high-amplitude permeabilization but allow for a protective depolarization step, which would prevent mitochondria from toxic calcium overload and oxidative stress.

### Limitations of the study

Our method allows to detect the non-selective mitochondrial membrane permeabilization as can be judged by the solute equilibration. However, it does not give specific information regarding the size of the PTP. Also, since mitochondrion becomes “invisible” following depolarization and permeabilization, it is impossible to follow its change in morphology after PTP activation. This limitation can be addressed in the future experiments by labeling organelle with the fluorescent tag that is maintained after PTP activation. In present study, we only use one of the methods to induce PTP. It will be interesting to investigate the relationship between depolarization and permeabilization using other Ca^2+^ ionophores and stress conditions that are known to involve PTP (e.g. oxygen-glucose deprivation).

## STAR★Methods

### Key resources table

**Table:** 

REAGENT or RESOURCE	SOURCE	IDENTIFIER
**Chemicals, peptides, and recombinant proteins**		

TMRM	Invitrogen	T668
Ferutinin	Sigma-Aldrich	SML1760; CAS: 00210302-17-3
Cyclosporin A	Sigma-Aldrich	C3662; CAS: 59865-13-3
FCCP	Sigma-Aldrich	C2920; CAS: 370-86-5
Fluo-4	Invitrogen	F14201

**Experimental models: Cell lines**		

Hap 1 Cells	He et al., 2017a	N/A
HAP 1 Δ (c+δ) Cells	He et al., 2017b	N/A
MEF Cells	Karch et al., 2019	N/A
MEF ANT Triple KO Cells	Karch et al., 2019	N/A

**Software and algorithms**		

Fiji ImageJ	Open Source	https://ImageJ.net/ImageJ
Ilastik	Open Source	https://github.com/ilastik/ilastik
Origin 2021b Software	OriginLab, Massachusetts USA	https://www.originlab.com/
Seahorse Wave Desktop Software	Agilent	https://www.agilent.com/

**Other**		

Seahorse XFe24 FluxPak	Agilent	102340–100

### Resource availability

#### Lead contact

Further information and requests for resources and reagents should be directed to and will be fulfilled by the lead contact, Maria Neginskaya (mn2452@nyu.edu).

#### Materials availability

This study did not generate new unique reagents.

### Experimental model and subject details

#### Cell lines

Immortalized HAP 1 and MEF cell lines were used for this study.^6^^,^^8^ HAP 1 cells were grown Iscove’s Modified Dulbecco’s Medium (IMDM), supplemented with 10% Heat-Inactivated Fetal Bovine Serum (HI FBS; Life Technologies), 10 mL per L of Antibiotic Antimycotic Solution (Penicillin/Streptomycin/Amphoterichin B; Sigma Aldrich) and 2 mM L-Glutamine. MEF cells were grown in high-glucose Dulbecco’s Modified Eagle Medium (DMEM; Cytiva) supplemented with 10% HI FBS, 10 mL per L of Antibiotic Antimycotic Solution, and 1X Non-Essential Amino acids (NEAA; Lonza). HAP 1 Δ (c+δ) cell line that lacks c and δ-subunits of ATP synthase was used for the study of the role of ATP synthase in high-conductance PTP. MEF ANT Triple KO cell line lacking 3 ANT genes was used to study the role of ANT in high conductance PTP. MEF ANT Triple KO cells were grown in the same media as MEF WT cells with the addition of 1mM Sodium Pyruvate (Gibco) and 25mg/500mL Uridine (Sigma Aldrich). Cells were maintained in a humidified cell incubator, at 37°C under a 5% CO2 atmosphere.

### Method details

#### Holographic and fluorescent imaging

The cells were plated on poly-D-lysine coated glass coverslips 24 h before imaging to reach the confluency of 70–90%. Before the experiment, the coverslips with the cells were placed in the imaging chamber and washed with Hank’s Balanced Salt Solution (HBSS, Gibco). TMRM fluorescent probe was used for estimation of mitochondrial membrane potential. Cells were incubated with 40 nM of TMRM for 15 min in room temperature in the darkness. Recording media contained 40 nM of TMRM. Ferutinin was used to induce calcium-induced PT. Minimal concentration of ferutinin that was able to reproducibly depolarize mitochondria was picked up for each cell type. RI images (holographic reconstructions) and TMRM signal were acquired every 15 s with aid of 3D Cell Explorerfluo (Nanolive, Switzerland) equipped with 60X objective. Protonophore FCCP (10 μM) was used at the end to observe the drop of membrane potential and normalize the TMRM signal.

We routinely monitored the change in cytoplasmic calcium along with mitochondrial depolarization after ferutinin additions using spinning disk microscope. For that cells were then incubated at room temperature in the darkness for 30 min in 500nM Fluo-4, followed by 15 min in 20nM TMRM. Cells were imaged every 10 s at 20x magnification, using a Nikon fluorescent microscope (Chiyoda, Tokyo, Japan) with a 488nm laser for Fluo-4 excitation and 561nm laser for TMRM excitation.

#### Seahorse assay

Analysis of mitochondrial functions in HAP 1 WT and HAP 1 Δ(c+δ) cells was performed on Seahorse XFe24 (Agilent Technologies, USA) (Nichols et al., 2017). Cells were plated on Seahorse XFe24 Cell culture 24-well microplates 24 h before experiment to reach the confluency 70–80% according to Agilent Technologies recommendations.

The night before the experiment, the cartridge containing the sensors was hydrated with 1 mL of XF Calibrant Solution per well and kept overnight in a CO2-free incubator. The day of the experiment, cells were washed with Seahorse XF DMEM medium that contained 1 mM pyruvate, 2 mM glutamine, and 10 mM glucose and incubated for 1 h in the CO2-free incubator (hypoxia). The cartridge was loaded with 30 μM of Ferutinin, 1 μM of FCCP and 0.5 μM of rotenone/antimycin A (Rot/AA) to the ports A, B and C accordingly to measure OCRs and ECARs. All the drugs were dissolved to the working concentrations using the Seahorse XF DMEM medium. Seahorse media, drugs and microplates were obtained from the Seahorse XFe24 Fluxpak.

Subsequently, the cells were loaded onto the analyzer and the measurements were conducted. The obtained data was exported and analyzed using the Seahorse Wave Desktop Software.

#### Holographic reconstruction processing

Fiji ImageJ was used to process holographic reconstructions. Multipage TIF files were prepared^19^ and plain RI images were reconstructed as a Z-stack maximal intensity projection from the volume of the cell that contained mitochondria. Ilastik, the interactive learning and segmentation toolkit, was used for mitochondrion segmentation. After being trained by the user, Ilastik tool creates the probability map of pixels that relate to mitochondria and based on the probability, classify them as mitochondria (Figure S6). Segmented images were converted to a binary image with Fiji “Make binary” tool. Resulted image is shown in Figure S6C.

#### Mitochondrial membrane permeabilization

To analyze the mitochondrial membrane permeabilization, we estimated the decrease in the RI of mitochondria by the decrease of the area occupied by mitochondria in reconstructed images. To do so, the regions of interest (ROIs) with functional mitochondria with maintained membrane potential were selected manually from the corresponding fluorescent images of cells labeled with TMRM (Figure S6D). These ROIs were used to estimate changes in membrane potential and applied to binary segmented masks created as described above (Figure S6C). Next, the area occupied by mitochondria was estimated in selected ROIs in each time frame. The decrease of the area indicated the decrease of mitochondrial RI and, thus, mitochondrial permeabilization (compare Figures S2A and S2B). Single mitochondrion tracking was performed manually by selecting the mitochondrion areas in RI images frame by frame. The same areas were used in corresponding TMRM fluorescent images to track the changes in mitochondrial membrane potential.

#### Quantification and statistical analysis

Origin 2021b software was used for data presentation, analysis and statistics. All the data presented as Mean ± SEM. The exact numbers of experiments (N) and cells (n) analyzed are mentioned in corresponding parts of the text. ANOVA and t-test were used to verify statistical significance (∗p < 0.05; ∗∗p < 0.01; ∗∗∗p < 0.001).

## Data Availability

• All data reported in this paper will be shared by the lead contact upon request. • This paper does not report original code. • Any additional information required to analyze the data reported in this paper is available from the lead contact upon request.
