# Supplementary material for: Both ANT and ATPase are essential for mitochondrial permeability transition but not depolarization
Source: iScience. 2022 Oct 28;25(11):105447. doi: 10.1016/j.isci.2022.105447 (PMC9647522; doi:10.1016/j.isci.2022.105447)
Supplement: Document S1. Figures S1–S6 [file mmc1.pptx]

## Slide 1
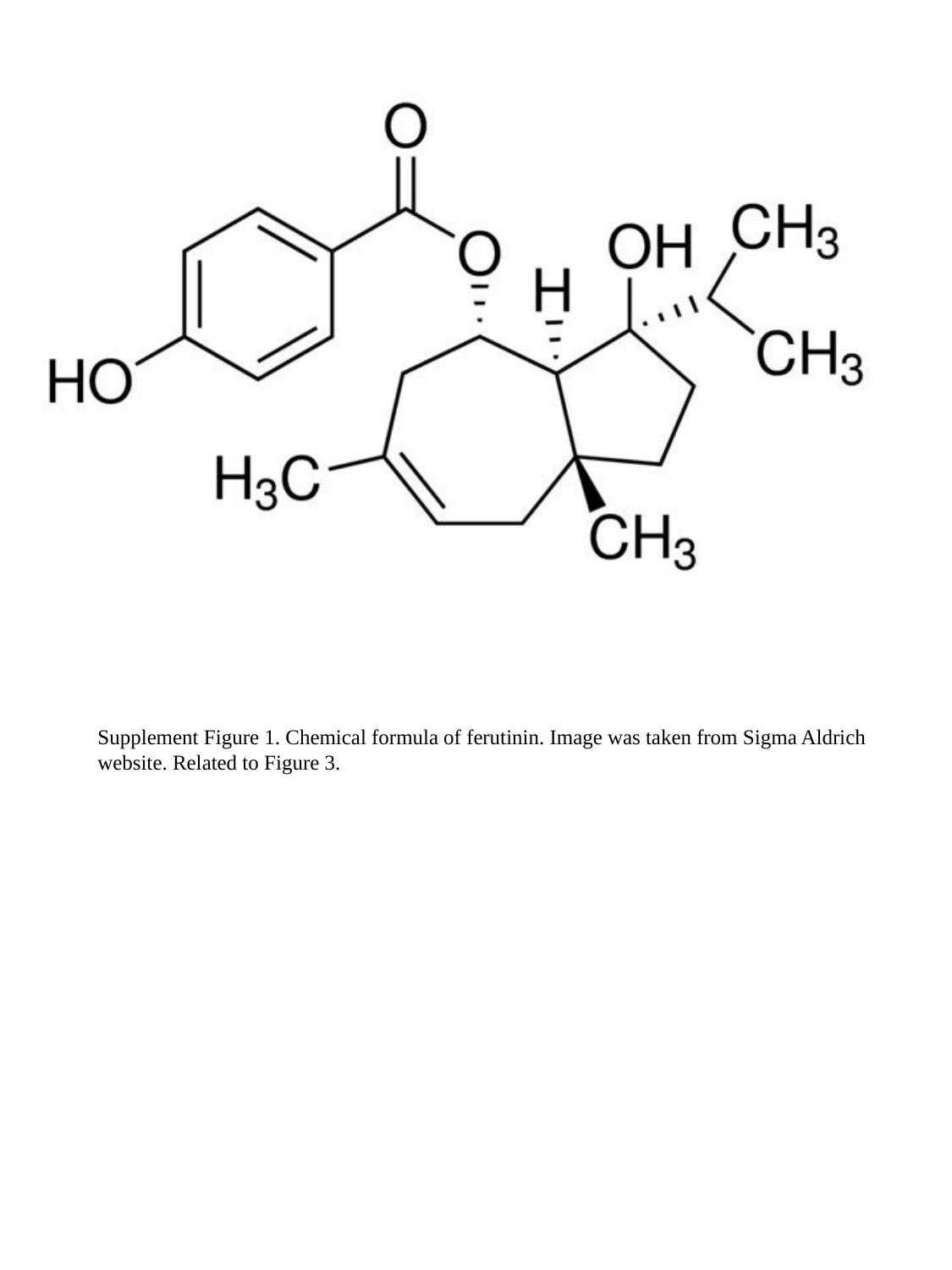

Supplement Figure 1. Chemical formula of ferutinin. Image was taken from Sigma Aldrich website. Related to Figure 3.

## Slide 2
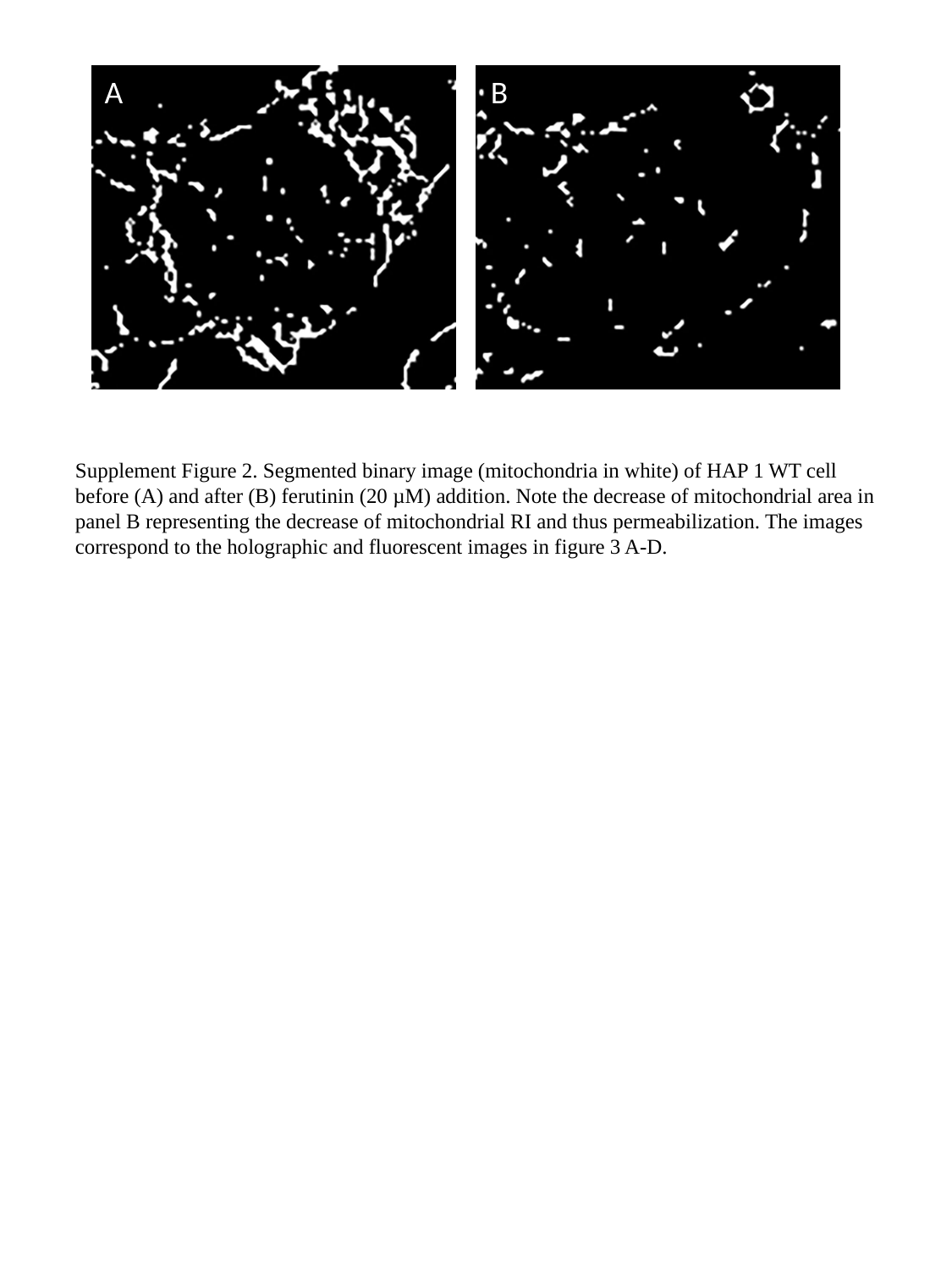

A
B
Supplement Figure 2. Segmented binary image (mitochondria in white) of HAP 1 WT cell before (A) and after (B) ferutinin (20 µM) addition. Note the decrease of mitochondrial area in panel B representing the decrease of mitochondrial RI and thus permeabilization. The images correspond to the holographic and fluorescent images in figure 3 A-D.

## Slide 3
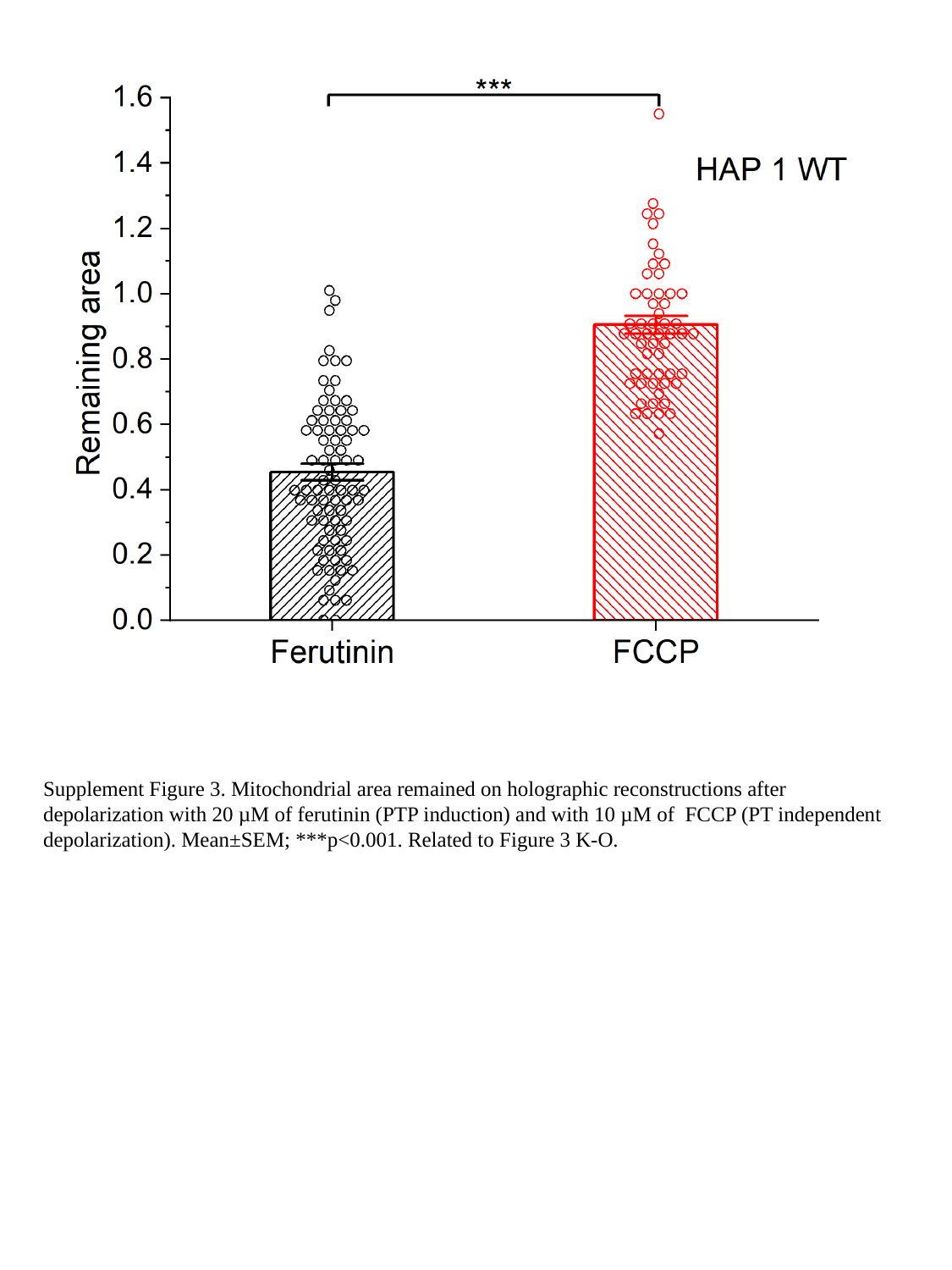

Supplement Figure 3. Mitochondrial area remained on holographic reconstructions after depolarization with 20 µM of ferutinin (PTP induction) and with 10 µM of FCCP (PT independent depolarization). Mean±SEM; ***p<0.001. Related to Figure 3 K-O.

## Slide 4
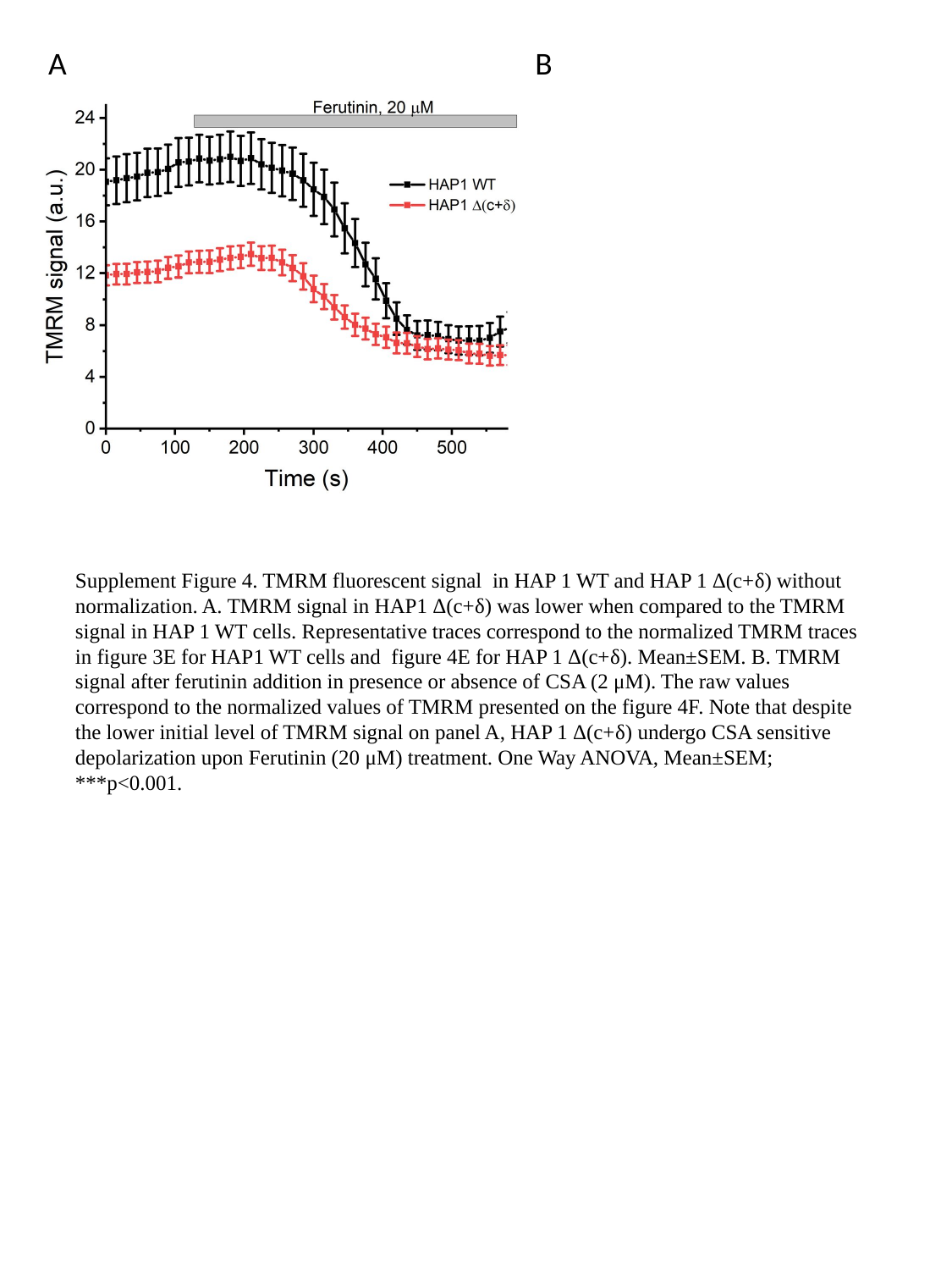

A
B
Supplement Figure 4. TMRM fluorescent signal in HAP 1 WT and HAP 1 Δ(c+δ) without normalization. A. TMRM signal in HAP1 Δ(c+δ) was lower when compared to the TMRM signal in HAP 1 WT cells. Representative traces correspond to the normalized TMRM traces in figure 3E for HAP1 WT cells and figure 4E for HAP 1 Δ(c+δ). Mean±SEM. B. TMRM signal after ferutinin addition in presence or absence of CSA (2 μM). The raw values correspond to the normalized values of TMRM presented on the figure 4F. Note that despite the lower initial level of TMRM signal on panel A, HAP 1 Δ(c+δ) undergo CSA sensitive depolarization upon Ferutinin (20 μM) treatment. One Way ANOVA, Mean±SEM; ***p<0.001.

## Slide 5
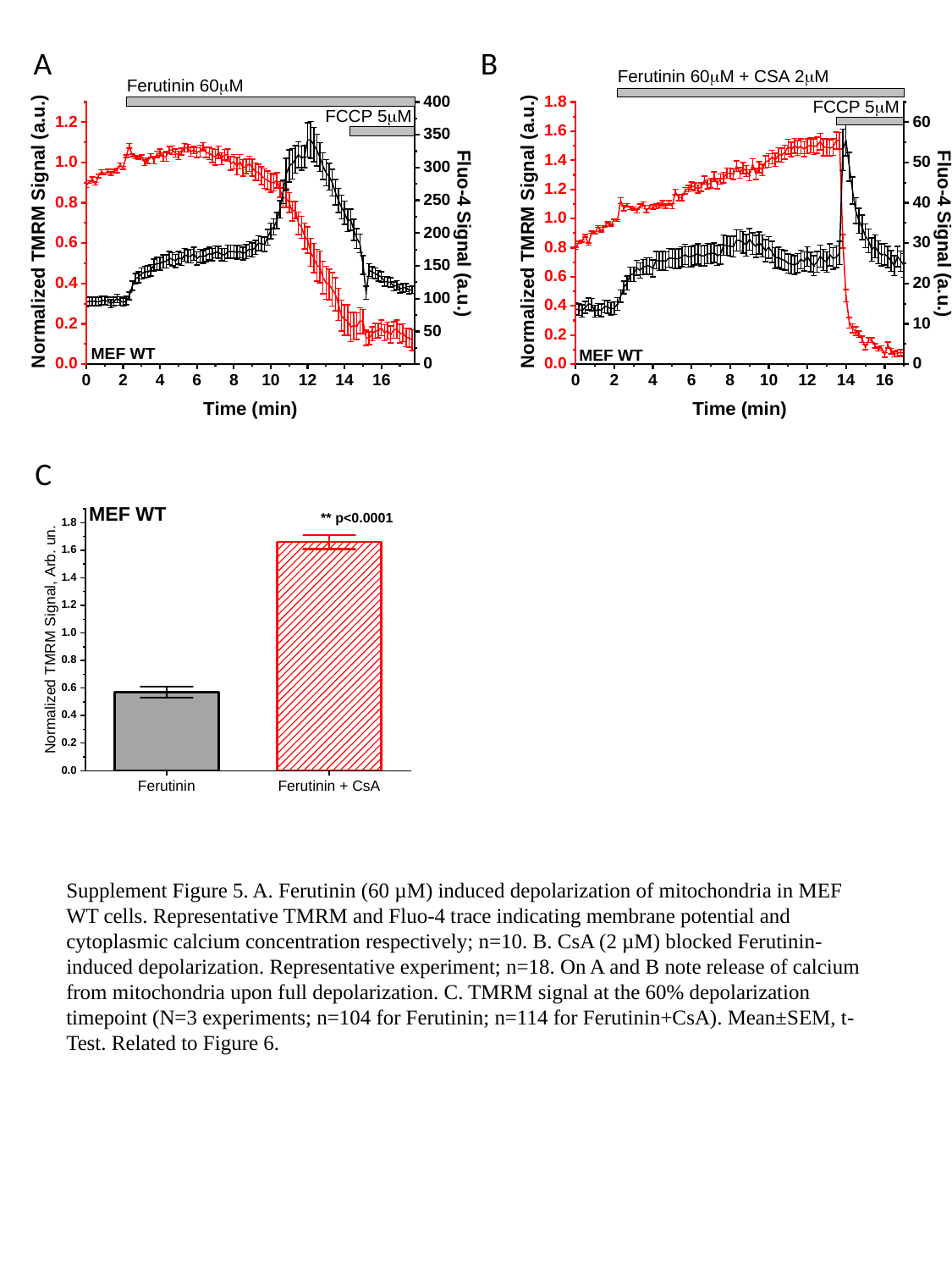

A
B
C
Supplement Figure 5. A. Ferutinin (60 µM) induced depolarization of mitochondria in MEF WT cells. Representative TMRM and Fluo-4 trace indicating membrane potential and cytoplasmic calcium concentration respectively; n=10. B. CsA (2 µM) blocked Ferutinin-induced depolarization. Representative experiment; n=18. On A and B note release of calcium from mitochondria upon full depolarization. C. TMRM signal at the 60% depolarization timepoint (N=3 experiments; n=104 for Ferutinin; n=114 for Ferutinin+CsA). Mean±SEM, t-Test. Related to Figure 6.

## Slide 6
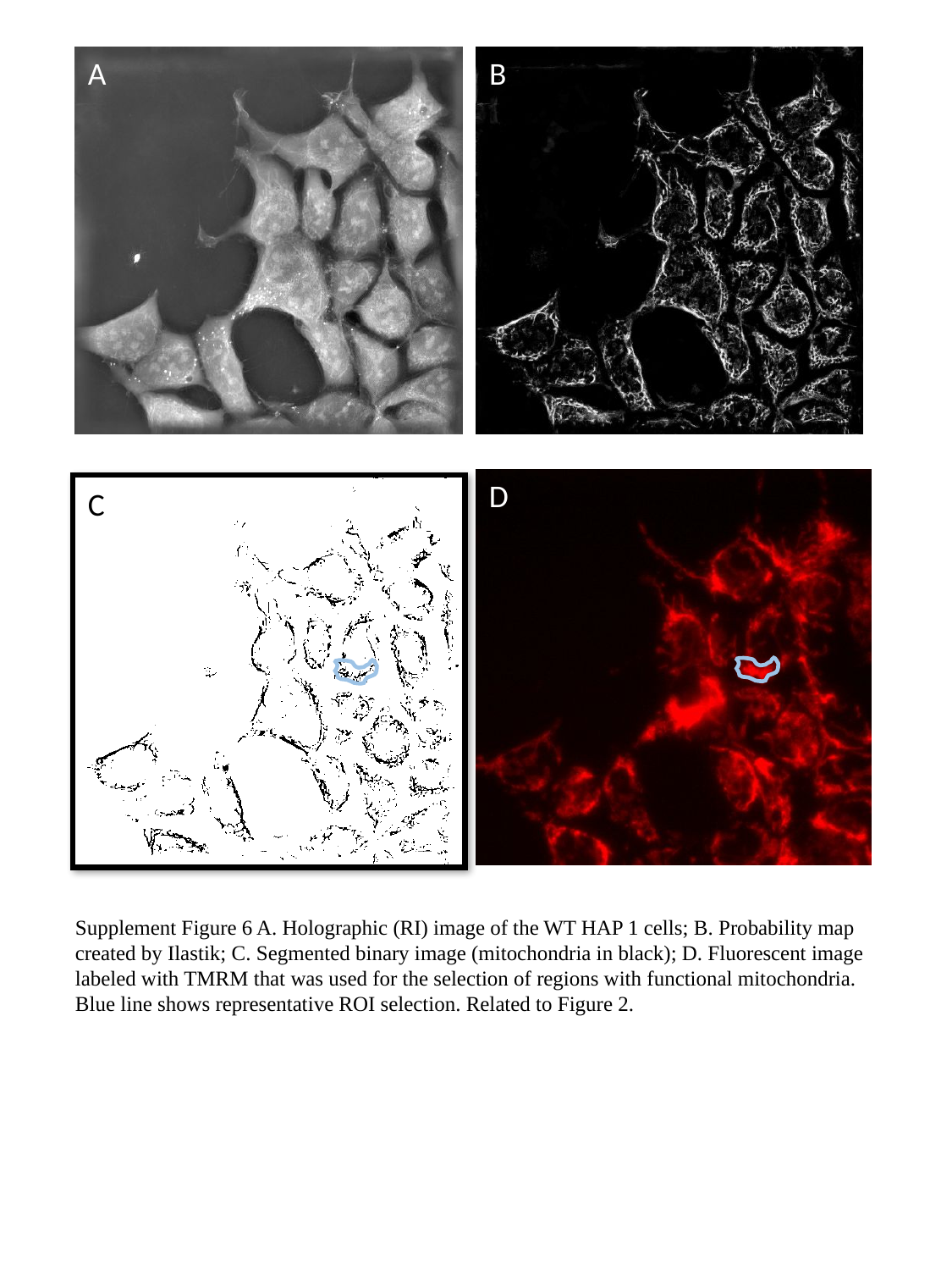

A
B
D
C
Supplement Figure 6 A. Holographic (RI) image of the WT HAP 1 cells; B. Probability map created by Ilastik; C. Segmented binary image (mitochondria in black); D. Fluorescent image labeled with TMRM that was used for the selection of regions with functional mitochondria.
Blue line shows representative ROI selection. Related to Figure 2.
